# Parenteral diclofenac infusion significantly decreases brain-tissue oxygen tension in patients with poor-grade aneurysmal subarachnoid hemorrhage

**DOI:** 10.1186/cc12714

**Published:** 2013-05-12

**Authors:** Alois J Schiefecker, Bettina Pfausler, Ronny Beer, Florian Sohm, Jan Sabo, Viktoria Knauseder, Marlene Fischer, Anelia Dietmann, Werner O Hackl, Claudius Thomé, Erich Schmutzhard, Raimund Helbok

**Affiliations:** 1 Department of Neurology, Neurological Intensive Care Unit, Innsbruck Medical University, Anichstrasse 35, Innsbruck, 6020, Austria; 2 Department of Neurosurgery, Innsbruck Medical University, Anichstrasse 35, Innsbruck, 6020, Austria; 3 UMIT: University for Health Sciences, Medical Informatics and Technology, Eduard Wallnoefer-Zentrum 1, Hall, 6060, Austria

## Abstract

**Introduction:**

Diclofenac, a nonsteroidal antiinflammatory drug, is commonly used as antipyretic therapy in intensive care. The purpose of this study was to investigate the effects of parenteral diclofenac infusion on brain homeostasis, including brain-tissue oxygen tension (P_b_tO_2_) and brain metabolism after aneurysmal subarachnoid hemorrhage (aSAH).

**Methods:**

We conducted a prospective, observational study with retrospective analysis of 21 consecutive aSAH patients with multimodal neuromonitoring. Cerebral perfusion pressure (CPP), mean arterial pressure (MAP), intracranial pressure (ICP), body temperature, and P_b_tO_2 _were analyzed after parenteral diclofenac infusion administered over a 34-minute period (20 to 45 IQR). Data are given as mean ± standard error of mean and median with interquartile range (IQR), as appropriate. Time-series data were analyzed by using a general linear model extended by generalized estimation equations (GEEs).

**Results:**

One-hundred twenty-three interventions were analyzed. Body temperature decreased from 38.3°C ± 0.05°C by 0.8°C ± 0.06°C (*P *< 0.001). A 10% decrease in MAP and CPP (*P *< 0.001) necessitated an increase of vasopressors in 26% (*n *= 32), colloids in 33% (*n *= 41), and crystalloids in 5% (*n *= 7) of interventions. P_b_tO_2 _decreased by 13% from a baseline value of 28.1 ± 2.2 mm Hg, resulting in brain-tissue hypoxia (P_b_tO_2 _<20 mm Hg) in 38% (*n *= 8) of patients and 35% (*n *= 43) of interventions. P_b_tO_2 _<30 mm Hg before intervention was associated with brain-tissue hypoxia after parenteral diclofenac infusion (likelihood ratio, 40; AUC, 93%; 95% confidence interval (CI), 87% to 99%; *P *< 0.001). Cerebral metabolism showed no significant changes after parenteral diclofenac infusion.

**Conclusions:**

Parenteral diclofenac infusion after aSAH effectively reduces body temperature, but may lead to CPP decrease and brain-tissue hypoxia, which were both associated with poor outcome after aSAH.

## Introduction

Fever is common in patients with aneurysmal subarachnoid hemorrhage (aSAH) and independently associated with poor outcome [1-4]. Brain-temperature elevations correlate with an increase of cerebral metabolic rate of oxygen (CMRO_2_) [5], intensified brain metabolism [6], aggravation of cerebral edema, and increased intracranial pressure (ICP) [7]. The hypothalamic set-point temperature can be lowered with nonsteroidal antiinflammatory drugs (NSAIDs) through inhibition of prostaglandine-E_2 _(PGE_2_) synthesis [8]. The NSAID diclofenac is commonly used in intensive care units for fever treatment [9-11]. Side effects of parenteral diclofenac, such as decrease of mean arterial blood pressure (MAP) [11] and cerebral perfusion pressure (CPP) [10,12], have been previously described. Hemodynamic stability for maintaining adequate cerebral perfusion during vasospasm is of utmost importance after aSAH [13]. The prognostic significance of low CPP and episodes of brain-tissue hypoxia (P_b_tO_2 _<20 mm Hg) after aSAH and traumatic brain injury (TBI) has been extensively studied [14-17]. The purpose of this trial was to describe the effects of parenteral diclofenac infusion on CPP, brain-tissue oxygen tension (P_b_tO_2_), and brain metabolism in the early period of aSAH.

## Materials and methods

### Patients

Prospectively collected data of 29 consecutive aSAH patients with brain multimodal neuromonitoring between September 2010 and March 2012 were retrospectively analyzed. Twenty-one patients receiving parenteral diclofenac infusion during the neuromonitoring time were included (see Additional file 1). Criteria for invasive neuromonitoring were approved by the institutional review board of Innsbruck Medical University (UN3898 285/4.8) as follows: (a) GCS ≤8, (b) poor likelihood for regaining consciousness within the following 48 hours, (c) high likelihood for surviving at least 48 hours, and (d) age older than 18 years. Written informed consent was obtained according to federal regulations.

### General management

In general, patient care conformed to guidelines of the American Heart Association [18]. Patients were clinically graded with the Hunt and Hess scale and radiologically assessed with the modified Fisher scale [19] of the first available cerebral computed tomography (CT) scan. Prophylactic, parenteral continuous nimodipine (1 to 2 mg/hour) was routinely given in all patients. A mean flow velocity >200 cm/second of the basal cerebral arteries assessed with transcranial Doppler sonography (TCD, DWL Doppler-Box system; Compumedics, Singen, Germany) was considered as TCD flow-velocity acceleration. Catheter angiography was performed in patients with TCD flow-velocity acceleration. Patients with angiographic vasospasm were treated with intraarterial nimodipine and vasopressors. All patients included in this study had continuous invasive blood pressure monitoring. Fluid therapy (colloids or crystalloids) were used as first-line, and vasopressors (noradrenaline, phenylephrine, or dobutamine) were used as second-line therapy for maintaining MAP and CPP. Whenever a critical CPP <50 mm Hg was reached, vasopressors were started/increased immediately. In case of unexpected blood pressure decreases, the parenteral nimodipine dose was decreased or stopped.

We did not perform wake-up trials on the patients, following our institutional guidelines. Therefore, delayed cerebral infarction (DCI) was defined as any new infarct appearing on cerebral CT scan that was judged by an independent radiologist to be attributable to vasospasm. Pneumonia was defined as radiologic infiltrate and elevated white blood cell count. Start and finish time of any pharmacologic intervention, including dosage, were exactly documented in the electronic patient data-management system (PDMS; Centricity Critical Care 7.0, General Electric Healthcare Company, IL, USA).

#### Fever definitions and management

Body temperature was measured with the temperature sensor of the bladder catheter. Fever was defined as a body temperature >38.4°C because this was the threshold for fever treatment. In certain cases, fever therapy was initiated at lower body temperatures at the discretion of the intensive care physician. Parenteral diclofenac (75 mg; maximum dosage, 150 mg/day) or parenteral acetaminophen (1,000 mg; maximum dosage, 3,000 mg/day) was used as first-line antipyretic therapy. The choice of treatment was at the discretion of the treating intensivist. In case of ineffectiveness or contraindications against the first-line antipyretic treatment (meperidine; Pethidine; 100 mg; maximum, 400 mg/day) as second-line therapy was given. If temperature still remained above 38.4°C, normothermia (36.5°C) was maintained in four patients with an invasive cooling device (CoolGard 3000 or ThermoGard XP; Alsius Corporation, Zoll Medical Corporation, MA, USA) as rescue therapy [20].

### Interventions

For fever treatment, 75 mg diclofenac-sodium diluted in 100 ml normal saline (Ratiopharm, parenteral diclofenac (Diclobene), 75 mg, drug approval number: 1-19719) was administered intravenously. Starting and ending time points of intervention were exactly documented in the PDMS. Interventions with administration of other antipyretics or invasive cooling 4 hours before or after parenteral diclofenac were excluded (details on the inclusion algorithm are shown in Appendix Figure 1).

**Figure 1:**
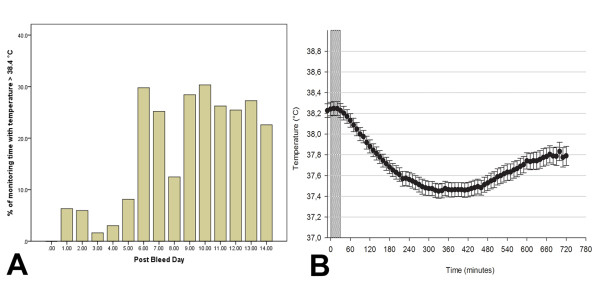
**Fever burden during monitoring time and body temperature after parenteral diclofenac**. **(A) **Cumulative fever burden in percentage of total monitoring time, fragmented on days after initial hemorrhage (post-bleed day 0-14). Time in fever (temperature >38.4°C) during monitoring time reached a maximum on post-bleed day 10. **(B) **demonstrating mean body temperature (●) after parenteral diclofenac (zero on X-axis is the first 10-minute-average-interval during intervention, *n *= 123; *P *< 0.001). The width of the dotted bar indicates median infusion time (34 minutes; 20 to 45 IQR). The maximum effect on core body temperature was reached after 330 minutes (median, 260 to 470, IQR). Values are illustrated in mean ± SEM.

### Monitoring and data acquisition

Monitoring probes were placed into the hemisphere deemed at greatest risk for secondary injury and either tunneled or fixed by using a triple-lumen bolt. CT scan of the brain was used to check the probe location, usually within 24 hours after implantation. P_b_tO_2 _was measured with a Clark-type probe (Licox, Integra, Germany), intracranial pressure (ICP) with an intraparenchymal probe (Neurovent, Raumedic, Germany). Brain-tissue hypoxia was defined as brain-tissue oxygen tension <20 mm Hg, based on previous studies [14,21] demonstrating increased odds for poor outcome and metabolic distress below this threshold value. Cerebral microdialysis (CMD) was performed by using a 100-kDa-cutoff microdialysis catheter (CMA-71; CMA/MicrodialysisTM, Stockholm, Sweden) at a perfusion rate of 0.3 μl/min. Samples were collected hourly and frozen at -80°C. Outcome was evaluated prospectively 3 months after aSAH with telephone interview by using the modified Rankin Scale (mRS). Poor neurologic outcome was defined as mRS >4. The study nurse was blinded to the monitoring data and their analysis. Complications (pneumonia, vasospasm, DCI) were stated after adjudication of all relevant clinical information in weekly meetings by the study team (RH, BP, RB, MF, AS, and ES).

### Data management and statistics

The PDMS was used to acquire digital data for ICP, MAP, CPP, P_b_tO_2 _, and body temperature every 3 minutes from the monitoring device (Carescape B650; General Electric Company, IL, USA). Monitoring data were averaged to 10-minute mean values. A 10-minute mean average before intervention was considered a baseline value for a hemodynamic parameter, and 1 hour before intervention was considered a baseline value for the CMD dataset. Time-series data were analyzed with a generalized linear model by using a normal distribution and identity link function. The model was extended by generalized estimating equations (GEEs) by using time after intervention as a factor and important parameters (CPP, body temperature) as covariates.

An autoregressive matrix of the first order (AR-1) was used to handle repeated observations within subjects [22]. Logarithmic transformation was applied to meet assumptions of normality. Cut-off levels were calculated by using receiver operating characteristics (ROCs).

SPSS 19.0 was used for statistical testing*. P *< 0.05 was considered statistically significant.

Data are given in median and interquartile range (IQR) or in mean ± one standard error of the mean (SEM).

## Results

### Study population and intervention

Baseline characteristics are demonstrated in Table 1. Mean age was 55 ± 11 years, and the median Hunt and Hess grade was 4 (3 to 5, IQR). Neuromonitoring was initiated at day 1 (1 to 2, IQR) and maintained for 12 days (8 to 14, IQR); 29% (*n *= 6) of patients had poor outcomes.

**Table 1:** Demographic details of study patients

Clinical characteristics			
	Age (years)		56 (47-63)
	Gender (female)		13 (62)
	Admission H&H	2-3	6 (29)
		4-5	15 (71)
	Admission Apache II Score		17 (14-19)
Admission radiologic characteristics			

	mFisher scale	1	2 (10)
		2	1 (4)
		3	9 (43)
		4	9 (43)
	IVH sum score		3 (0-6)
	Aneurysm size (mm)		9 (4-11)
	Generalized cerebral edema		10 (48)
	Intracerebral hematoma		7 (33)
Surgical procedures			

	Hydrocephalus requiring EVD		18 (86)
	Clipping		14 (67)
Complications			

	Pneumonia		13 (62)
	DCI		4 (19)
	Moderate to severe angiographic vasospasm		6 (29)
Outcome characteristics			

	Length of hospital stay (days)		40 (29-48)
	Three-months mRS	0-1	5 (24)
		2-3	7 (33)
		4-5	5 (24)
		6	4 (19)

Apache II score (acute physiology and chronic health score); DCI, delayed cerebral infarction; EVD, external ventricular drainage; H&H, Hunt and Hess grade; IVH, intraventricular hemorrhage; mFisher scale, modified Fisher scale; mRS, modified Rankin scale. Data are presented in median (IQR) and count (%).

One-hundred twenty-three interventions were analyzed. A median of four interventions (two to eight, IQR) per patient were administered over a 34-minute period (20 to 45, IQR). Mean central venous pressure at baseline was 13 ± 0.2 mm Hg. Ten days (7 to 12 days, IQR) after aSAH was the median time point of intervention, when fever was most common (30% of daily monitoring time; Figure 1A).

### Effects of parenteral diclofenac infusion on body temperature

Body temperature at baseline (38.3 ± 0.05°C) decreased by 0.8 ± 0.06°C to a minimum value of 37.5 ± 0.05°C within 330 minutes (260 to 470, IQR) after the start of an intervention (*P *< 0.001; Figure 1B). In 9% (*n *= 11) of interventions, temperature did not decrease after parenteral diclofenac. We found a significant interaction between body temperature and MAP (*P *= 0.02), but no interaction between body temperature and P_b_tO_2_.

### Effects of parenteral diclofenac infusion on MAP, CPP, and ICP

**Figure 2:**
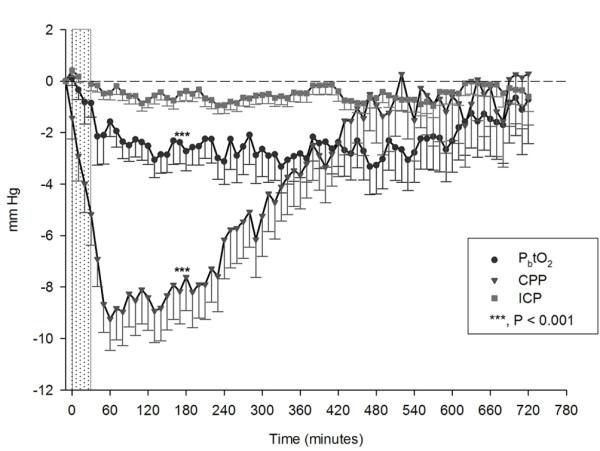
**Changes from mean baseline values after parenteral diclofenac (zero on X-axis is the first 10-minute-average interval during interventions; *n *= 123)**. CPP (▼, baseline = 85 ± 1.4 mm Hg; *P *< 0.001), ICP (■, baseline, 8.6 ± 0.4 mm Hg); and P_b_tO_2 _(●, baseline = 28.1 ± 2.2 mm Hg; *P *< 0.001). The dotted bar illustrates the median diclofenac infusion time (34 minutes; IQR, 20 to 45). Values are presented in mean ± SEM. CPP, cerebral perfusion pressure; ICP, intracranial pressure; IQR, interquartile range; P_b_tO_2_, brain-tissue oxygen tension. Initial CPP decrease after parenteral diclofenac occurred within the first 10 minutes, indicated as point zero at the X-axis.

MAP (baseline, 93 ± 1.2 mm Hg) and CPP (baseline, 85 ± 1.4 mm Hg) decreased by 10% after intervention (*P *< 0.001; Figure 2). Maximum blood pressure decreases were observed 160 minutes (90 to 320, IQR) after intervention. Colloids were administered in 33% (*n *= 41) and crystalloids in 5% (*n *= 7) 29 minutes (20 to 86, IQR) after intervention. Vasopressors were increased within 75 minutes (30 to 155 minutes, IQR) in 26% (*n *= 32) of interventions. A decrease of CPP <70 mm Hg and <50 mm Hg was observed in 71% (*n *= 87) and 12% (*n *= 15) of interventions, respectively. ICP (baseline: 8.6 ± 0.4 mm Hg) did not significantly change after intervention.

### Effects of parenteral diclofenac infusion on P_b_tO_2_

**Figure 3:**
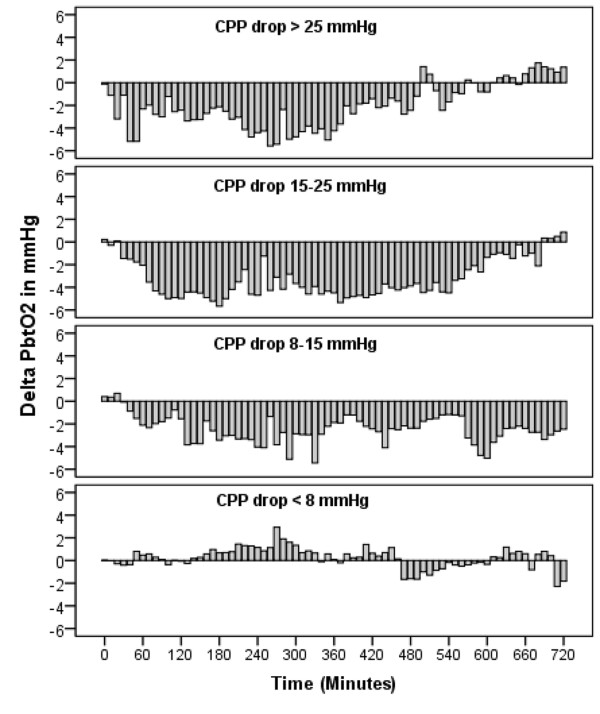
**Time-locked changes in P_b_tO_2 _from baseline of four different CPP groups (quartiles) representing maximum CPP decrease observed during the study period**.

**Figure 4:**
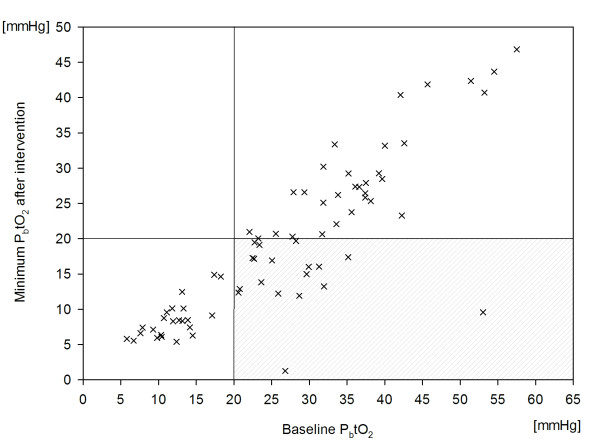
**Correlation between P_b_tO_2 _(x) reaching minimum values after parenteral diclofenac and P_b_tO_2 _values before intervention**. Lines represent P_b_tO_2 _threshold of 20 mm Hg. The right lower quadrant indicates P_b_tO_2 _values ≥20 mm Hg at baseline reaching hypoxic values after intervention.

P_b_tO_2 _decreased by 13% from a baseline value of 28.1 ± 2.2 mm Hg to 24.5 ± 2.1 mm Hg (*P *< 0.001; Figure 2), resulting in brain-tissue hypoxia (P_b_tO_2 _<20 mm Hg) in 35% (*n *= 43) of all interventions and in 38% (*n *= 8) of patients. This finding remained significant (*P *< 0.01) after adjusting for CPP and body temperature, indicating an intrinsic effect of diclofenac on P_b_tO_2_. An interaction occurred between P_b_tO_2 _and CPP (*P *= 0.02), which is shown in Figure 3. Baseline P_b_tO_2 _<30 mm Hg (Figure 4) was associated with brain-tissue hypoxia after parenteral diclofenac (likelihood ratio, 45; specificity, 81%; sensitivity, 88%; *P *< 0.001; OR 85.1; 95% CI, 13 to 550), with an area under the curve (AUC) of 93% (95% CI, 87% to 99%). Brain-tissue hypoxia was observed 3 hours (1.5 to 3 hours, IQR) after intervention and persisted for 25 minutes (18 to 180 minutes, IQR). A CPP decrease <70 mm Hg was associated with brain-tissue hypoxia (*P *< 0.01).

The percentage of total monitoring time with P_b_tO_2 _<20 mm Hg (32 ± 9.4% in patients with good outcome versus 66% ± 12% in patients with poor outcome: OR = 1.04; 95% CI, 1.001 to 1.08; *P *< 0.05), but not the absolute time (48 ± 20 hours versus 56 ± 27 hours; *P *= 0.07) was independently associated with poor outcome after adjusting for disease severity.

### Effects of parenteral diclofenac infusion on cerebral metabolism

**Figure 5:**
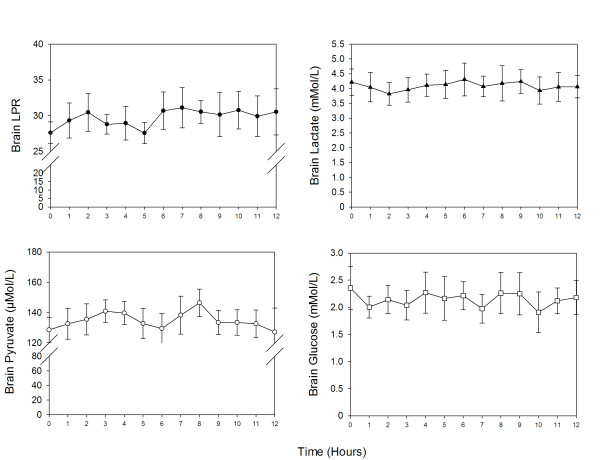
**Changes in brain metabolism time locked to parenteral diclofenac therapy (hours from intervention)**. Values presented in mean ± SEM.

Cerebral microdialysis (CMD) was available in 48% (*n *= 10) of patients and 28% (*n *= 34) of interventions. No significant change in brain metabolism was noted after parenteral diclofenac (Figure 5) when compared with baseline values (CMD-lactate, 4 ± 0.3 m*M*; CMD-pyruvate, 141 ± 7 μ*M*; CMD-LPR, 28 ± 1.5; CMD-glucose, 2 ± 0.2 m*M*; and CMD-glutamate, 22 ± 8 μ*M*).

## Discussion

Our main findings are that parenteral diclofenac infusion after aSAH is associated with a significant decrease of CPP and brain-tissue oxygen tension. P_b_tO_2 _values <30 mm Hg before intervention were highly predictive of consecutive brain-tissue hypoxia (P_b_tO_2 _<20 mm Hg). The percentage of time in brain-tissue hypoxia was independently associated with poor outcome.

P_b_tO_2 _reflects the balance between oxygen supply and demand and can be used as a surrogate marker for cerebral blood flow (CBF) [23,24]. The ability to maintain adequate CBF relatively independent of changes in CPP is termed normal cerebral autoregulation. Impaired cerebral autoregulation frequently occurs after aSAH and may result in a direct dependence of P_b_tO_2 _on CPP [25]. We did not assess cerebral autoregulation, but found a decrease in CPP related to the decrease in P_b_tO_2_. This mechanism may well explain the P_b_tO_2 _decrease in our patients; especially, a CPP <70 mm Hg was associated with brain-tissue hypoxia after intervention. This finding is in line with a recent study indicating a higher risk for brain-tissue hypoxia at CPP values <70 mm Hg [14].

P_b_tO_2 _is dependent not only on oxygen delivery, but also on cerebral oxygen consumption [23]. Cerebral metabolic rate of oxygen (CMRO_2_) as an indicator of cerebral oxygen consumption is temperature dependent [5]. Experimental studies showed a reduction of CMRO_2 _after parenteral NSAID injection [5]. Therefore, one might expect an increase of P_b_tO_2 _due to reduced CMRO_2 _after temperature reduction.

Interestingly, P_b_tO_2 _remained decreased even after CPP had recovered to baseline values. One explanation for this observation could be the inhibitory effect of diclofenac on the cyclooxygenase (COX) [26]. PGE_2_-syntheses is COX dependent and plays an important role in maintaining CBF and cerebral autoregulation [27]. Inhibition of PGE_2 _synthesis by diclofenac may reduce CBF and therefore P_b_tO_2_. Another explanation for this prolonged P_b_tO_2 _decrease may be neurovascular coupling, a mechanism of the brain adapting the CBF to the cerebral ener-gy demand [27]. A decrease in body temperature might reduce cerebral energy demand and therefore CBF [5,28]. This hypothesis is furthermore supported by the lack of metabolic changes despite a decrease in P_b_tO_2_.

We found a significant interaction between mean arterial blood pressure and body temperature after diclofenac intervention. This is in line with previously described hemodynamic changes related to physiological mechanisms of temperature regulation, including vasodilatation, sweating, and inhibition of muscle activity [29].

Hemodynamic changes after parenteral diclofenac necessitated increased use of vasopressors and parenteral fluid therapy. Adrenergic stress can promote the development of myocardial stunning [30], which may increase the risk of cerebral infarction from vasospasm, hypotension, and pulmonary edema [31]. Diclofenac infusion time was not standardized, which could have influenced hemodynamic side effects in this study. A randomized controlled trial by Cormio *et al. *[12] investigated continuous low-dose diclofenac infusion (0.004 to 0.08 mg/kg BW/h) for fever management after TBI and aSAH. The authors showed that this treatment regimen effectively decreases body temperature without the occurrence of hemodynamic side effects. In their control group, antipyretic therapy (0.2 mg/kg diclofenac, 1 g acetaminophen) was administered over a 30-minute period. This infusion rate was associated with a significant CPP decrease (*P *= 0.03) and is comparable to our results. These findings suggest an association of hemodynamic side effects with the application time of antipyretic therapy. Further studies are needed to investigate whether continuous low-dose diclofenac should preferably be used in aSAH patients.

We found an effective reduction of body temperature after parenteral diclofenac, whereas the degree of temperature reduction and the duration to maximum efficacy were in line with the literature [10,12] and pharmacologic properties of diclofenac (half-life, 1 to 2 hours) [32].

This study has several limitations. First, a causal relation between parenteral diclofenac and P_b_tO_2 _decrease cannot be certainly proven based on our data. Importantly, all continuous physiologic and pharmacologic data as well as intervention time points were exactly recorded with an electronic chart and patient-management system. Second, this observational study has no power to compare the effects of different antipyretic drugs on P_b_tO_2_, which can be demonstrated only by randomized controlled trials. Third, the infusion rate was not standardized, which might bias the study results. Fourth, we did not analyze cerebral autoregulation or CBF, and microdialysis data must be interpreted cautiously because of limited sample size with simultaneous P_b_tO_2 _recordings available during interventions.

Nonetheless, this is the first study investigating brain-tissue oxygen tension and cerebral metabolism after parenteral diclofenac in poor-grade aSAH patients.

## Conclusions

This study indicates that parenteral diclofenac effectively reduces body temperature, but may lead to CPP decline and brain-tissue hypoxia, which are both associated with poor outcome after SAH. Tight monitoring and awareness about possible hemodynamic side effects seem mandatory when using parenteral diclofenac in patients with poor-grade aSAH.

## Key messages

• Tight monitoring of hemodynamic side effects is mandatory when using parenteral diclofenac in patients with poor-grade aSAH.

• Parenteral diclofenac after poor-grade aSAH effectively reduces body temperature, but may decrease MAP, CPP, and brain-tissue oxygen tension.

• P_b_tO_2 _<30 mm Hg before parenteral diclofenac was highly predictive of brain-tissue hypoxia (P_b_tO_2 _<20 mm Hg) after intervention.

## Abbreviations

aSAH: aneurysmal subarachnoid hemorrhage; AR-1: autoregressive matrix of the first order; AUC: area under the curve; CBF: cerebral blood flow; CMD: cerebral microdialysis; CMRO_2: _cerebral metabolic rate of oxygen; COX: cyclooxygenase; CPP: cerebral perfusion pressure; CT: computed tomography; DCI: delayed cerebral infarction; GCS: Glasgow Coma Scale; GEE: generalized estimation equation; ICP: intracranial pressure; IQR: interquartile range; MAP: mean arterial pressure; mRS: modified Rankin Scale; NSAID: nonsteroidal antiinflammatory drug; OR: odds ratio; P_b_tO_2: _brain-tissue oxygen tension; PDMS: patient data-management system; PGE_2_: prostaglandin-E_2; _ROC: receiver operating characteristic; SEM: standard error of mean; TBI: traumatic brain injury; TCD: transcranial Doppler sonography.

## Competing interests

The authors declare that they have no competing interests.

## Authors' contributions

AS was involved in the acquisition of data, statistical analysis, interpretation of data, study design, writing and manuscript drafting. RH was involved in the study design, interpretation of data, statistical analysis, manuscript writing and drafting, and final revision of the manuscript. ES, RB, BP, JS, MF, AD, and VK participated in the acquisition and interpretation of data and in the final revision of the manuscript. FS and CT were involved in the study design and data acquisition. WH was involved in study design, data processing, and statistical analysis. All authors read and approved the final manuscript.

## Supplementary Material

Additional file 1 **Flow chart of patients and interventions included in the study**. This file contains a flow chart of patients and interventions included in or excluded from the study. Click here for file
