# Supplementary figures and images for: Parenteral diclofenac infusion significantly decreases brain-tissue oxygen tension in patients with poor-grade aneurysmal subarachnoid hemorrhage
Source: Crit Care. 2013 May 12;17(3):R88. doi: 10.1186/cc12714 (PMC3706816; doi:10.1186/cc12714)

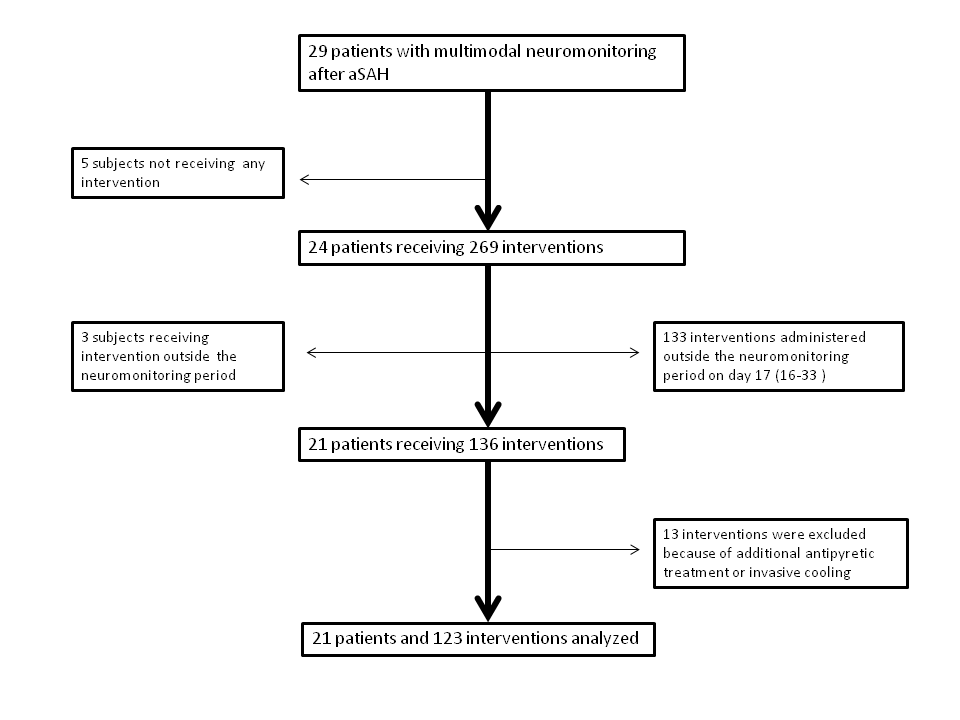


Appendix figure 1: Flow chart of patients and interventions included/excluded in the study.

Supplement: Additional file 1 — Flow chart of patients and interventions included in the study. This file contains a flow chart of patients and interventions included in or excluded from the study. [file cc12714-S1.DOC]
